# Potential Immunotherapeutic Targets on Myeloid Cells for Neurovascular Repair After Ischemic Stroke

**DOI:** 10.3389/fnins.2019.00758

**Published:** 2019-08-09

**Authors:** Ziyu Zhu, Li Zheng, Yan Li, Tingting Huang, Yu-Chieh Chao, Lijun Pan, Hui Zhu, Yanhua Zhao, Weifeng Yu, Peiying Li

**Affiliations:** ^1^Department of Anesthesiology, Renji Hospital, School of Medicine, Shanghai Jiaotong University, Shanghai, China; ^2^Department of Radiology, Renji Hospital, School of Medicine, Shanghai Jiaotong University, Shanghai, China

**Keywords:** stroke, neurovascular unit, brain repair, myeloid cell, microglia, macrophage, neutrophil

## Abstract

Neurological deficits and cognitive dysfunctions caused by acute ischemic stroke pose enormous burden to the stroke families and the communities. Restoration of the normal function of the neurovascular unit following ischemic stroke is critical for improving neurological recovery and cognitive functions after stroke. Recent evidence suggests that the myeloid cells including both the resident microglia and infiltrating monocytes/macrophages and neutrophils are highly plastic in response to the environmental cues. They intimately interact with multiple components of the neurovascular unit in response to the alarmins, danger associated pattern molecules (DAMPs) and other signals released from the ischemic brain. The aim of this review is to discuss the reciprocal interactions between the myeloid cells and the ischemic neurovascular unit during the late repair phase of cerebral ischemic stroke. We also summarize potential immunotherapeutic targets on myeloid cells and new therapeutic approaches targeting myeloid cells, such as cell transplantation, mitochondrial dynamic and extracellular vesicles-based therapy et al to enhance neurovascular repair for better stroke recovery.

## Introduction

The neurovascular unit (NVU) is composed by neurons, endothelial cells, pericytes, smooth muscle cells, astrocytes, microglia and extracellular matrix components. It maintains brain homeostasis of the brain (Banks et al., 2018; Morrison and Filosa, 2019). All the components of NVU interact with each other to maintain the neuronal milieu that is required for proper neuronal functioning by balancing energy, preserving the integrity of the blood brain barrier (BBB), releasing neurotrophic factors, uptake and recycling of neurotransmitters and et al. (Halassa et al., 2007; Charveriat et al., 2017). In response to cerebral ischemic stroke, the integrity of the BBB compromises leading to leakage of harmful blood components into the CNS, immune cell infiltration, and aberrant transport and clearance of molecules. All these changes contribute to the dysfunction of the NVU which is associated with long term neurological impairments (Fisher and Saver, 2015; Villaseñor et al., 2017). The reconstruction of NVU and the remodeling of neuronal circuitry turns out as an essential player in long-term neurological recovery after stroke (Gurer et al., 2009; Lake et al., 2017). However, the repair of NVU is a complex process involving clearing of neuronal debris, neurogenesis, angiogenesis, establishing the new neuronal circuitry, controlling neuroinflammation and et al. (Lake et al., 2017; Zhao Z. et al., 2017), thus it remains as a big challenge to promote the NVU repair after ischemic brain injury.

After ischemic stroke, both glial cells and peripheral immune cells can be activated by a variety of mechanisms, including neuronal “help me” signals, like lipocalin-2 (Xing et al., 2014; Blochet et al., 2018), interleukin 4 (IL-4) (Zhao et al., 2015), and a complex mixture of extracellular proteins functioning as damage associated molecular patterns (DAMPs), such as high-mobility group box 1 (HMGB1), hypoxia-inducible factor 1α (HIF-1α), S100B and et al. (Chamorro et al., 2012; Fu et al., 2015; Li et al., 2018b). Notably, myeloid immune cells are gaining increasing attention due to their unique function in anti-inflammation, clearing cellular debris and promoting neuronal plasticity (Iadecola and Anrather, 2011; Shichita et al., 2014; Fu et al., 2015; Zhao S. C. et al., 2017; Wang X. et al., 2018).

Myeloid cells are blood cells that arise from a large heterogeneous multipotent stem cell population, the hematopoietic stem cells (HSC) lineage (Kondo et al., 1997; Akashi et al., 2000) which resides in the bone marrow and possess the ability to give rise to diverse cell types in the immune system and the blood, including granulocytes, monocytes, erythrocytes and platelets (Paul et al., 2015). Some myeloid populations can develop directly from yolk sac progenitors without apparent bone marrow intermediates, such as tissue resident macrophages (Kondo et al., 1997; Akashi et al., 2000; Shibata and Suzuki, 2017). Although peripheral monocyte-derived macrophages and microglia can both be developed from the same yolk sac progenitors, they should not be considered as one cellular population in the injured brain (Greenhalgh et al., 2018). After injury, monocyte-derived macrophages can enter the brain and directly communicate with microglia and suppress the microglia-mediated phagocytosis and inflammation after spinal cord injury (Greenhalgh et al., 2018). In the central nervous system (CNS), the main tissue resident macrophages including perivascular macrophages (PVM), meningeal macrophages (MM), and choroid plexus macrophages (CPM) (Henning et al., 2009) and microglia migrate into the CNS during early neural development depending on transcription factor Pu.1 and Irf8 (Hanisch and Kettenmann, 2007; Kierdorf et al., 2013).

In the recent decades, microglia and monocytes/macrophages are intensively studied in the context of cerebral ischemic brain injury (Hu et al., 2012; Garcia-Culebras et al., 2018), with compelling evidence emerging, thus we mainly focused on these cells in this review. After cerebral ischemia, both residential microglia and macrophages can be activated within 24 h and the infiltration of monocyte-derived macrophages and activation of microglia peaks within 3–4 days after stroke onset (Lalancette-Hebert et al., 2007; Gelderblom et al., 2009; Wattananit et al., 2016). Polymorphonuclear neutrophil granulocytes (PMNs) are another myeloid cell population that can infiltrate into the ischemic brain and affect ischemic brain injury (Frijns and Kappelle, 2002). These innate immune cells play important roles in determining the ischemic infarcts. For example, PMNs release matrix metallopeptidase 9 (MMP-9) which degrades the BBB and exacerbates ischemic brain injury (Li et al., 2013a) and tissue-plasminogen activator related hemorrhagic transformation (Mao et al., 2017). Selective microglia elimination disturbed neuronal calcium responses, increased calcium overload and increased the incidence of spreading depolarization, thus significantly increased the infarct size by 60% (Szalay et al., 2016). Depletion of monocyte/macrophages in cerebral ischemic stroke reduced hemorrhagic infarct transformation or reduced brain injury in monocyte/macrophage depleted stroke mice (Gliem et al., 2012; Ma et al., 2016). However, there’s also a recent study showed no impact on stroke outcome with monocyte/macrophages depleted (Schmidt et al., 2017).

Recent researches have highlighted that the impact of these myeloid cells on the ischemic brain injury largely depend on different phenotypes at different stages of stroke (Kurisu et al., 2018). As it’s well-known that microglia and macrophages can be divided into different phenotypes depending on their functions on the inflammatory responses (Mantovani et al., 2013; Guruswamy and ElAli, 2017). Classically-activated (M1) microglia/macrophages usually secret pro-inflammatory cytokines, such as inducible nitric oxide synthase (iNOS), tumor necrosis factor α (TNFα), interleukin 23 (IL-23), interleukin 1β (IL-1β), interleukin 12 (IL-12) and et al. (Mills et al., 2000; Guruswamy and ElAli, 2017; Li et al., 2017), while alternatively activated (M2) microglia/macrophages are characterized by their production of anti-inflammatory cytokines, such as interleukin 10 (IL-10) and transforming growth factor β (TGF-β) (Gordon and Taylor, 2005; Guruswamy and ElAli, 2017) and they can be polarized with IL-4 via a signal transducer and activator of transcription 6 (STAT6)-dependent pathway (Stein et al., 1992; Gao et al., 2015). Recent studies further identified more specific subpopulation of M2 microglia/macrophages, such as M2a, M2b, M2c, and Mox (Hu et al., 2015). Importantly, the phenotypes and functions of these cells are highly dynamic after ischemic injury (Tsuyama et al., 2018). Likewise, PMNs may also have different phenotypes in different stages after ischemic stroke, a pro-inflammatory so called N1 and an anti-inflammatory N2 phenotype (Fridlender et al., 2009).

However, the terminology of microglial/macrophage polarization was questioned and believed to hinder research progress (Ransohoff, 2016). There are far more phenotypes of microglia/macrophages than M1/M2, such as amyotrophic lateral sclerosis specific microglia (Chiu et al., 2013), phagocytic monocyte-derived macrophages (Yamasaki et al., 2014), immunosuppressive CD11c^+^ microglia and et al. (Kan et al., 2015). There are also cases that microglia/macrophage may concurrently display both pro- and anti-inflammatory phenotypes (Morganti et al., 2016). In addition, transcriptomics and genomic studies reveal that the function of microglia are versatile beyond immune responses, such as synaptic modulation and neurotrophic support (Wes et al., 2016). Single-cell RNA-sequencing gene profiling showed that macrophages in traumatic brain injury are not comprised of distinctly polarized cell subsets, instead, these macrophages are uniquely and broadly activated (Kim et al., 2016). Therefore, it is not rigorous to classify microglia/macrophages imprudently into M1/M2 classifications (Ransohoff, 2016).

**FIGURE 1:**
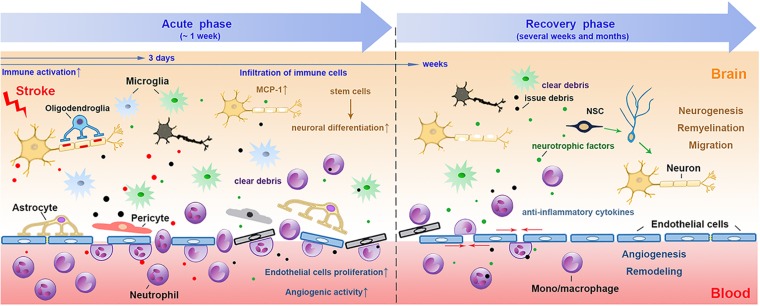
The involvement of myeloid immune cells during the NVU repair after cerebral ischemic stroke. In the acute phase of ischemic stroke, the damaged associated signals from the ischemic brain activate resident and peripheral myeloid immune cells, such as microglia, peripheral mono/macrophages and neutrophils. These cells penetrate into the CNS through the disrupted BBB from 1 day after stroke, peaking at 2–3 days and may last for several weeks. The phagocytic function of myeloid cells enables them to remove tissue debris in both acute and repair stages. During the recovery phase after stroke, myeloid cells with strong plasticity could differentiate into different phenotypes and release several neurovascular nutritional factors, which may enhance neurovascular regeneration and remodeling up to weeks and months after stroke. NVU, neurovascular unit; CNS, central nervous system; BBB, blood brain barrier; NSC, neural stem cells.

Recently, compelling evidence is suggesting the role of the distinct “healing” myeloid cells during the restoration of NVU after stroke, including phagocytosis of damaged neuronal debris, promoting neurogenesis and angiogenesis, re-establishment of the neuronal circuitry, improving white matter repair and et al. during different phases after stroke (Figure 1).

In this review, we will summarize the distinct functions of different subsets of myeloid cells during the “repair” of NVU discuss the identified mechanisms underlying their restoration of the NVU after stroke.

## Divergent Functions of the “Healing” Myeloid Cells During NVU Repair After Ischemic Stroke

### Phagocytosis of Damaged Tissue Debris After Stroke

**FIGURE 2:**
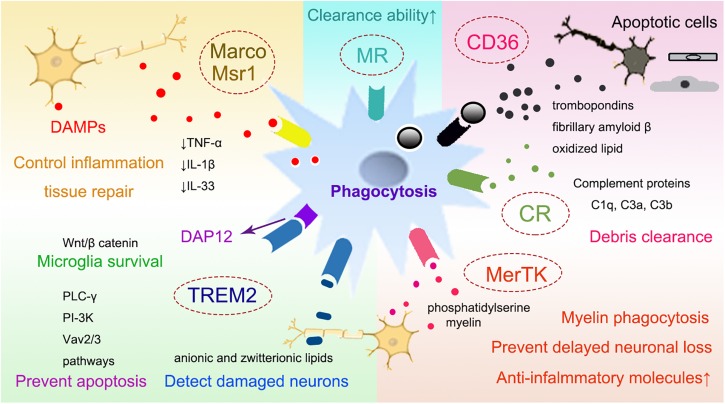
The receptors that mediate phagocytosis of myeloid immune cells. Several receptors have been suggested to mediate the phagocytosis of myeloid cells, such as the class A scavenger receptor Marco and Msr1, the class B scavenger receptor CD36, TREM2, CRs, MRs, and MerTK. Myeloid cells can phagocytose the DAMPs released from damaged neurons with the receptors such as Msr1 and Marco, which subsequently downregulate the neurovascular inflammation and enhance brain repair. Through CD36, myeloid cells can also clear tissue debris from apoptotic cells, thrombospondins, oxidized lipid, et al. TREM2 is important for preventing microglia apoptosis, it also promotes the survival of microglia via the Wnt/β catenin pathway. Complement receptor, such as CR1 and CR3, may bind to apoptotic neuronal cells and contribute to clearing debris and limiting neuroinflammation. MerTK is associated with the myeloid phagocytosis of myelin, neurons and eryptotic erythrocytes, leading to prevent delayed neuronal loss after stroke. CD36, cluster of differentiation 36; TREM2, triggering receptor expressed on myeloid cells 2; CRs, complement receptors; MRs, mannose receptors; MerTK, the mer receptor tyrosine kinase; DAMP, danger associated molecular pattern; Msr1, macrophage scavenger receptor 1; Marco, macrophage receptor with collagenous structure.

By removing necrotic tissues, myeloid cells may accelerate brain repair by enhanced phagocytosis after intracerebral hemorrhage (ICH) (Wan et al., 2016; Fumagalli et al., 2019). Using structured illumination microscopy, a super-resolution technique which helps study phagocytosis, it was shown that myeloid cells were encompassed with potent phagocytic abilities once they infiltrated into the ischemic brain (Fumagalli et al., 2019). There are several different receptors expressed on microglia/macrophages that mediate the phagocytosis of these cells, including class A, class B scavenger receptors, the surface receptor triggering receptor expressed on myeloid cells-2 (TREM2), complement receptors (CRs) (Dustin, 2016), mannose receptors (Galea et al., 2005) and the mer receptor tyrosine kinase (MerTK) (Healy et al., 2016, 2017). In addition, purinergic receptors, such as P2Y12 receptors as key regulators of microglia process extension, also contribute to their responses to brain injury (Kluge et al., 2017; Fukumoto et al., 2018). We will briefly summarize the phagocytic aspects of some recently studied receptors after stroke (Figure 2).

#### The Class A Scavenger Receptors

The class A scavenger receptor includes macrophage scavenger receptor 1(Msr1) and macrophage receptor with collagenous structure (Marco). Msr1 is also known as CD204. Both of these receptors are important for the clearance of DAMPs, including peroxiredoxins, high mobility group box 1 protein and S100 calcium-binding protein A8/9 from the ischemic brain (Milne et al., 2005; Shichita et al., 2017). Msr1 is up-regulated in infiltrating mononuclear phagocytes from day 1 to day 3 after stroke onset and is dependent on the transcription factor Mafb, which plays an important role in the regulation of lineage-specific hematopoiesis (Shichita et al., 2017). Combined Msr1 and Marco deficiency results in sustained inflammation, leading to impaired clearance of DAMPs and ultimately an enlarged infarct volume and exacerbated neurological deficits (Shichita et al., 2017). The expression level of Msr1 helps to distinguish the repairing macrophages from inflammatory ones, since Msr1^hi^ macrophages express less inflammatory cytokines (TNF-α, IL-lβ, and IL- 23) and are instead the major sources of insulin-like growth factor-1(IGF-1), a neurotrophic factor (Shichita et al., 2017).

#### The Class B Scavenger Receptor Cluster of Differentiation 36 (CD36)

The class B scavenger receptor CD36, is a highly glycosylated integral membrane protein expressed in microglia and monocytes/macrophages (Cho et al., 2005; Woo et al., 2016). With high affinity toward apoptotic cells and many ligands such as thrombospondins (TSPs), fibrillary amyloid β and oxidized lipids, CD36 has been suggested to play a pivotal role in the clearance of cell debris during the recovery phase in post-stroke brains (Cho et al., 2005; Woo et al., 2016; Stamova et al., 2018). Stroke induces increased mRNA expression of CD36 and TSP-1/2 mRNA levels in the ipsilateral hemisphere both during the acute and recovery phases (Woo et al., 2016). Deletion of CD36 results in poorer outcome due to lack of attenuation of nuclear factor-κB (NF-κB) mediated inflammation and diminished removal of apoptotic neuronal debris (Woo et al., 2012). CD36 deficiency or SS-31, a cell permeable tetra-peptide known to down-regulate CD36 pathway, significantly attenuated monocyte chemoattractant protein 1 (MCP-1), C-C chemokine receptor type 2 (CCR2) mRNA up-regulation and injury size in the transient ischemic stroke (Kim et al., 2015). Increased expression of CD36 expression in monocyte/macrophages has been associated with higher phagocytic indices in the brain immune cells after ischemic stroke (Woo et al., 2016).

#### The Surface Receptor TREM2

The surface receptor TREM2 is expressed exclusively on myeloid cells, including brain microglia (Hakola, 1972; Nasu et al., 1973). It was first identified as a genetic cause of neurodegenerative disease called Nasu-Hakola disease [or polycystic lipomembranous osteodysplasia with sclerosing leukoencephalopathy (PLOSL)] (Hakola, 1972; Nasu et al., 1973). It is characterized as axonal degeneration and white matter loss, as well as cortical atrophy in clinical patients (Sasaki et al., 2015). TREM2 binds anionic carbohydrates, anionic bacterial products and various phospholipids (Daws et al., 2003; Cannon et al., 2012). It transmits intracellular signals through the associated transmembrane adapter DNAX activation protein of 12 kDa (DAP12), an immune-receptor tyrosine-based activation motif (ITAM)-bearing adapter molecule that transduces activating signals in natural killer (NK) and myeloid cells (Peng et al., 2010). DAP 12 can recruit the protein tyrosine kinase Syk, which phosphorates phosphoinositide phospholipase C-γ (PLC-γ), phosphoinositide 3-kinases (PI3Ks) and Vav2/3 to induce the mobilization of intracellular calcium and the reorganization of actin and prevent apoptosis (Peng et al., 2010). TREM2 also stabilizes β-catenin by inhibiting its degradation via the Akt/GSK3β signaling pathway, thus promotes microglial survival (Zheng et al., 2017). TREM2 expression has been associated with phagocytic activities of microglia in cerebral ischemia and Alzheimer’s disease (AD) (Kawabori et al., 2015; Wu et al., 2017; Kurisu et al., 2018; Wei et al., 2019). Loss of TREM2 results in reduced phagocytosis of a variety of substrates, including apoptotic neurons (Hsieh et al., 2009), amyloid plaques and anionic and zwitterionic lipids on damaged neurons (Wang et al., 2015; Yuan et al., 2016). Using bone marrow chimeric mice, it has been shown that TREM2 on the brain microglia is more essential to post-stroke recovery compared to those on circulating macrophages (Kurisu et al., 2018).

#### CRs in Myeloid Cells

The complement system is emerging as an important player of cerebral ischemic brain injury. A variety of complement proteins have been shown to be induced after cerebral ischemic stroke, such as C1q, C3a and C3b (Fumagalli et al., 2019). It has been shown that C1q can bind to apoptotic neuronal cells and produce opsonizing C3b and C4b fragments, which are responsible for phagocytosis of apoptotic cells. Furthermore, C1q also binds to IgM, pentraxin-3 and serum amyloid P component during the C1q-mediated removal of apoptotic cells. C1q^–/–^ mice exhibited impaired elimination of immune complexes and were more susceptible to neuroinflammation (Orsini et al., 2014). There are several CRs that have been linked to the phagocytic function of myeloid cells, such as CR1 and CR3 (Dustin, 2016). CR1 is a membrane-bound glycoprotein and is a receptor for C3b and C4b. It has been shown that the expression of CR1 on microglia was increased after lipopolysaccharide (LPS) induction and played a detrimental role in neuronal death after LPS stimulation or in the pathology of AD. However, the role of CR1 in the context of ischemic stroke remains unknown. CR3, also known as CD11b/CD18 or Mac-1(Ma et al., 2019) is a member of the integrin β2 subfamily. It is deeply involved in the innate immune responses and also the induction of phagocytosis by interacting with its ligand iC3b. The phagocytosis mediated by C3b is usually beneficial for clearing debris, apoptotic or necrotic neurons and limiting neuroinflammation after brain injury (Rotshenker, 2003; Fourgeaud and Boulanger, 2007).

#### Mannose Receptors (MRs)

The MRs, also known as cluster of differentiation 206, CD206, are C-type lectin which can express on microglia/macrophages, dendritic cells and et al. The extracellular region of MRs include an N-terminal cysteine-rich domain that is able to bind sugars with high affinity, such as mannose and fructose, thus scavenging unwanted high mannose N-linked glycoproteins from the circulation (Lee et al., 2002). The expression of MRs has been shown to increase after cerebral ischemic stroke in the phagocytic microglia/macrophages within the ischemic boundary (Giraldi-Guimaraes et al., 2012). Induction of increased MRs expression is believed to enhance the clearance ability of microglia/macrophages and thus provide neuroprotection against ischemic stroke (Giraldi-Guimaraes et al., 2012).

#### Tyrosine Kinase Receptor, MerTK

MerTK is a member of tyrosine kinase receptor family that plays important role in mediating phagocytosis by myeloid cells. The expression of MerTK is correlated with the myelin phagocytosis of myeloid cells not only in multiple sclerosis (Healy et al., 2016, 2017), but also the phagocytosis of ischemic neurons after cerebral ischemia (Neher et al., 2013). After ischemic stroke, the expression MerTK was transiently upregulated. Inhibition of MerTK may prevent delayed neuronal loss after stroke (Neher et al., 2013). In the model of ICH, loss of MerTK reduced hematoma clearance and exacerbated iron deposition, thus worsened neurological recovery (Chang et al., 2018). Therefore, MerTK is a receptor that is associated the phagocytosis of a variety targets, such as myelin, neurons and erythrocytes.

Collectively, there are multiple phagocytic receptors expressed on myeloid cells that are promising in clearing the debris and promoting brain repair after stroke. However, the roles and the underlying mechanisms of some phagocytic receptors remain largely unknown in the context of cerebral ischemia and thus warrant further investigation.

### Anti-inflammatory Effect of Myeloid Cells and Brain Repair After Stroke

Mounting studies have suggested that the alternatively activated or anti-inflammatory phenotype of microglia/macrophages are characterized in their anti-inflammatory effects, with the signature proteins expressed, such as arginase-1, CD206 or macrophage MR, Ym1 and TGF β and IL-10 (Gordon and Taylor, 2005; Hu et al., 2015; Miro-Mur et al., 2016; Ge et al., 2017; Gaceb et al., 2018). Several mechanisms have been suggested to induce the anti-inflammatory effect of microglia and macrophages, such as the activation of chemokine (C-X3-C motif) ligand 1-CX3C chemokine receptor 1 (CX3CL1-CX3CR1) signaling, IL-33/ST2 signaling and IL-4/peroxisome proliferator-activated receptor γ (PPARγ) signaling (Mizuno et al., 2003; Lyons et al., 2009; Liu X. et al., 2016; Yang et al., 2017). High expression of endogenous CX3CL1 in neurons acts as an “on” signal to stop the quiescent state of microglia and leads to a tonic activation of CX3CR1 on microglia and acts as a neuronal “off” signal maintaining microglia in a quiescent state (Biber et al., 2007; Eyo and Wu, 2013). CX3CR1^+^Ly6C^low^ “patrolling” monocytes may play a role in repair processes in the ischemic brain (Garcia-Bonilla et al., 2016). However, there are also studies showing that Ly6C^hi^ monocytes are protective in acute ischemic stroke by promoting macrophage polarization (Chu et al., 2015). IL-4 was able to induce peroxisome proliferator-activated receptor γ (PPARγ) activation thereby enhanced long-term functional recovery of cerebral ischemic stroke (Zhao et al., 2015). ST2, a member of the IL-1 receptor family, and its ligand interleukin-33(IL-33) play critical roles in immune regulation and inflammatory responses. ST2 deficiency aggravates neurological deficits up to 7 d after transient middle cerebral artery occlusion (Yang et al., 2017). The IL-33/ST2 signaling potentiates the expression of IL-10 and other anti-inflammatory genes in primary microglia and stimulates the production of IL-10 from microglia, which, in turn, enhances neuronal survival after ischemic insult (Yang et al., 2017).

Recent evidence suggests that similar to the concept of classically activated and alternatively activated macrophages, neutrophils also have pro-inflammatory and anti-inflammatory phenotypes, called N1 and N2 phenotypes (Fridlender et al., 2009; Cuartero et al., 2013). Modulation of PPARγ with rosiglitazone results PMN to shift toward a more pronounced N2-phenotype after stroke (Cuartero et al., 2013). Notably, the anti-inflammatory N2 neutrophils were found to limit excessive immune response, promoting neuronal survival and successful brain remodeling after ischemic stroke and ICH (Hermann et al., 2018). Interleukin-27/lactoferrin is suggested to modulate neutrophil functions toward a “beneficial” phenotype in the treatment of ICH (Zhao X. et al., 2017).

Given the compelling evidence suggesting the association of anti-inflammatory phenotype of these myeloid cells with brain repair after stroke and ICH, the roles and mechanisms of the myeloid cell associated NVU repair remain to be further explored. Furthermore, it was recently demonstrated that lack of myeloid lineage cell autophagy could aggravate the inflammation after cerebral ischemic stroke, suggesting a conflicting role of myeloid cells in the neuroinflammation after stroke (Kotoda et al., 2018). In general, neuroinflammation is a complex and constantly evolving cellular process. However, we need to be cognizant of the fact that we still do not know which aspects of neuroinflammation are beneficial and which are detrimental. It is not by chance that there are no successful anti-inflammatory therapies to treat stroke patients, not least due to the risks surrounding immunosuppression (Smith et al., 2015; Siniscalchi et al., 2016), bringing other side effects like depression (Wium-Andersen et al., 2017).

### Promoting Neurogenesis and Angiogenesis

#### Neurogenesis

Neurogenesis is a key step toward recovery from cerebral ischemic stroke (Sun et al., 2018). It is a natural process that occurs in the adult hippocampus everyday as was shown by a previous study measuring the concentration of nuclear-bomb-test-derived ^14^C in genomic DNA (Spalding et al., 2013). After stroke, neurogenesis can be induced in the ipsilateral subventricular zone (SVZ) 1 to 2 weeks after the ischemic insult and cell proliferation returns to baseline 6 weeks later (Thored et al., 2006). In addition, stroke also induces neuroblasts to differentiate into neurons (Arvidsson et al., 2002) or to elicit a neurogenic program in striatal astrocytes in mice (Magnusson et al., 2014).

Microglia possess the ability to support neurogenesis after ischemic stroke through pleiotropic mechanisms (Ziv et al., 2006; Kettenmann et al., 2011). During 2, 6 and 16 weeks after middle cerebral artery occlusion, there are more activated microglia migrating from the ipsilateral SVZ to the striatum together with neuroblasts in rats (Thored et al., 2009). These microglia exhibit pro-neurogenic phenotypes with increased expression of IGF-1, which can promote proliferation and differentiation of neural stem cells (NSCs) (Thored et al., 2009) and contribute to neural repair and survival after ischemic injury (Zhu et al., 2009). Removal of proliferating microglia by ganciclovir treatment decreases the level of neurotrophic IGF1, leading to the exacerbation of ischemic neuronal damage and deteriorated stroke outcomes (Lalancette-Hebert et al., 2007). Therefore, microglia are believed to have a predominantly repairing function in promoting the survival of neurons in the resolution phase of ischemic brain injury (Szalay et al., 2016).

Brain-derived neurotrophic factor (BDNF) is another important neurotropic protein that can be produced by microglia after stroke (Cook et al., 2017; Verma et al., 2017). It has been shown to regulate synaptic function and promote multiple learning tasks performance (Coull et al., 2005; Parkhurst et al., 2013). The micro-environment of microglia/macrophage is critical for their pro-neurogenic properties. Microglia activated by IL-4 and interferon γ (IFNγ) promote neurogenesis, while LPS activated microglia block neurogenesis in hippocampal slice cultures (Butovsky et al., 2005; Liu et al., 2013). Modulation the polarization of microglia/macrophages with chronic metformin treatment is associated with enhanced angiogenesis and neurogenesis after cerebral ischemic stroke (Jin et al., 2014). Meningeal macrophage polarization induced by BCG vaccination is helpful for the neurogenesis and spatial cognition (Qi et al., 2017). In the neuron-macrophage co-culture, it has been shown that molecular factors secreted by macrophages can promote neurite outgrowth (Yun et al., 2018).

#### Angiogenesis and Vascular Remodeling

**FIGURE 3:**
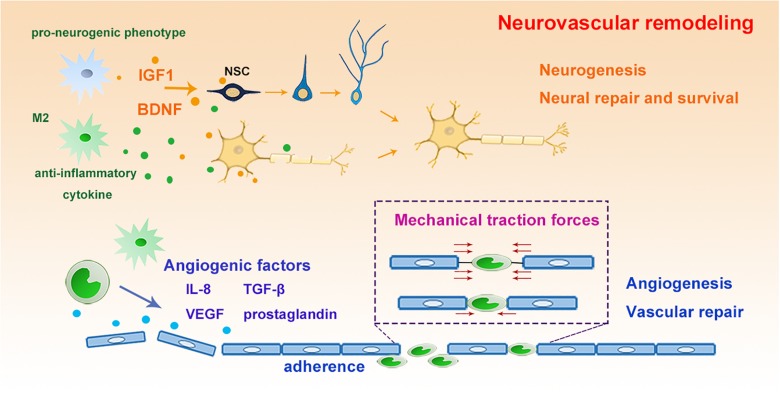
The roles of myeloid cells for neurovascular remodeling. Microglia switch into pro-neurogenic phenotype and anti-inflammatory phenotype with increased release of IGF-1, BDNF and anti-inflammatory cytokines, which promote the proliferation and differentiation of NSCs, leading to enhanced neurogenesis, neural repair and NSC survival and differentiation. Furthermore, myeloid cells express angiogenic factors as IL-8, VEGF et al to promote vascular repair. Moreover, they could generate mechanical traction forces to pull the injured endothelial ends, rebuilding the ligation to enhance repair. IGF, insulin-like growth factor; BDNF, brain derived neurotrophic factor; NSC, neural stem cells; IL-8, interleukin 8; VEGF, vascular endothelial growth factor.

Remodeling of the cerebral vasculature after cerebral ischemic stroke involves angiogenesis and also the growth of new blood vessels from pre-existing vascular tree, both of which ultimately promotes the re-establishment of the microcirculation and the blood supply to the damaged brain tissue (Lapi and Colantuoni, 2015). Vascular remodeling can last for more than 3 weeks after cerebral ischemia. It has been associated with improved neurological outcome after stroke (Lapi and Colantuoni, 2015). Vascular endothelial growth factor (VEGF) and endothelium-released nitric oxide are important factors in the generation of new vessels and formation of new vasculature during microvascular remodeling (Lapi and Colantuoni, 2015). Monocytes/macrophages can express interleukin-8 (IL-8), VEGF, TGF-β, prostaglandin, and MMP-9, all of which can enhance the angiogenic process after stroke (Sanberg et al., 2010). In addition to secreting angiogenic factors, macrophages can also repair the ruptured vascular mechanically (Liu C. et al., 2016). Using i*n vivo* time-lapse imaging in zebrafish, it has been shown that macrophage arrive at the cerebral vascular rupture lesion and extend their filopodia or lamellipodia to physically adhere to both endothelial ends (Liu C. et al., 2016). Furthermore, these macrophages can promote the ligation and the repair of the rupture by generating mechanical traction forces to pull the endothelial ends (Liu C. et al., 2016; Figure 3).

### Re-establishment of the Neuronal Circuit by Enhancing Axonal Sprouting and Synaptic Plasticity

**FIGURE 4:**
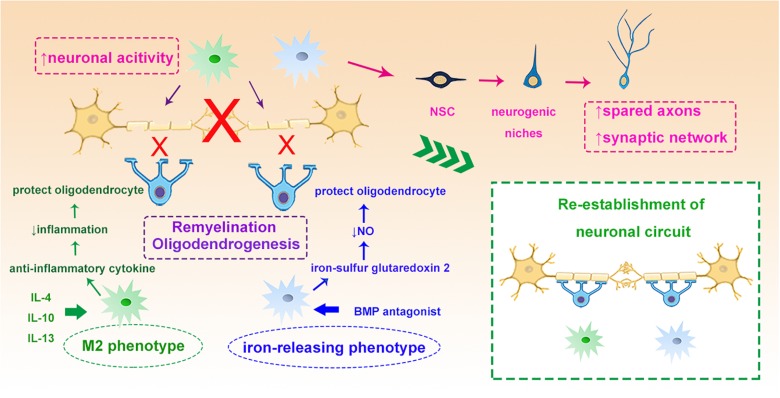
Myeloid cells and neuronal circuit re-establishment. Myeloid cells such as microglia can modify neuronal activity and stimulate the NSCs to sprout axons and rebuild synaptic networks. IL-4 induced M2 myeloid cells and BMP antagonist induced iron-releasing microglia protect oligodendrocytes through different mechanisms, such as enhancing remyelination and oligodendrogenesis, which all contribute to the re-establishment of the neuronal circuit. NSC, neural stem cells; IL-4, interleukin 4; BMP, bone morphogenetic protein.

To achieve functional recovery after stroke, the newly generated or differentiated neurons need reorganization and rewiring in the ischemic lesion. These remodeling processes are often associated with the sprouting of spared axons and modifying the synaptic networks to establish new circuits (Chu et al., 2015). Interestingly, emerging evidence is suggesting that microglia can rapidly modify neuronal activity, modulate synaptic function and promote the integration of NPCs from endogenous neurogenic niches into functional networks after brain injury (Wake et al., 2013; Sandvig et al., 2018). In addition, recent evidence suggests that microglia/macrophages play an important role in the remyelination through protecting oligodendrocytes, augmenting oligodendrogenesis and promoting oligodendrocyte progenitor cell (OPC) differentiation both in stroke and other neurodegenerative diseases (Miron et al., 2013; Moore et al., 2015; Hagemeyer et al., 2017). By regulating the phenotypic switch of microglia with IL-4 or inducing iron-releasing phenotype with noggin, a bone morphogenetic protein (BMP) antagonist, the “healing” capacity toward remyelination of microglia can be significantly enhanced (Shin et al., 2018). Administration of iron-sulfur glutaredoxin 2, the oxidoreductase, inhibits peroxynitrite formation thus reduces the nitric oxide release from activated microglia and protects oligodendrocytes from myelin damage in experimental autoimmune encephalomyelitis (Lepka et al., 2017). Activation of microglia with IL-13 or IL-10 not only induces generation of OPCs from the SVZ after focal demyelination in the corpus callosum (Naruse et al., 2018), but also enhances the survival and differentiation of these cells during CNS remyelination (Miron et al., 2013; Figure 4).

## Potential Therapeutic Strategies Targeting Myeloid Cells in the Treatment of Ischemic Stroke

### Bone Marrow Derived Stem Mononuclear Cells Transplantation

Bone-marrow derived mononuclear cells (MNCs) based therapy is gaining increasing attention because of the pleotropic effects in improving neurological recovery after stroke (Brenneman et al., 2010; Vahidy et al., 2016; Yang et al., 2016). Interestingly, with increased expression of IL-10, IL-6, MCP-1, VEGF, and TNF-α, post-stroke MNCs treatment leads to a better recovery on neurological testing and reduces lesion size as compared to pre-stroke MNCs (Yang et al., 2012). In 2016, thirty-nine patients with subacute ischemic cerebral infarct due to large artery occlusion in the middle cerebral artery (MCA) territory were recruited to receive bone marrow mononuclear (BMMN) stem cell transplantation. The study suggests that it is safe to transplant autologous BMMN stem cell intra-arterially in subacute MCA ischemic stroke patients, but unfortunately, no significant improvement of motor, language disturbance, or infarction volume was detected in the stem cell-treated group as compared to the non-treated group within an observation period of 12 months (Deierborg et al., 2010).

### Adoptive Transfer of Monocytes/Macrophages

It’s recently proposed that preconditioned monocytes/macrophages may play an endogenous protective role after ischemic brain injury (Garcia-Bonilla et al., 2018). Depletion of circulating monocytes/macrophages or selective targeting of CCR2 in bone marrow-derived cells exacerbated the BBB disruption and thus caused delayed clinical deterioration and hemorrhagic conversion of the infarctions after stroke (Gliem et al., 2012). On the other hand, transfer of splenic monocytes isolated from preconditioned mice into naive mice 7 h after transient MCAO reduced ischemic brain injury (Garcia-Bonilla et al., 2018). Mechanistically, gene expression and functional studies showed that IL-10, inducible nitric oxide synthase, and CCR2 in monocytes are essential for the neuroprotection (Garcia-Bonilla et al., 2018). Interestingly, a clinical study with 13 stroke patients and 13 case-control stroke patients showed that intrathecal injection of autologous M2 macrophages in stroke patients is safe and may lead to improved neurological recovery (Chernykh et al., 2016). Although the study is still preliminary with a small sample size, it opens up a new direction toward myeloid-cell based stroke therapy to protect the ischemic brain.

Nevertheless, when it comes to immune-cell based therapy, it’s always important to consider the condition of the recipients. For example, in our previous study, we found adoptive transfer of regulatory T cells or *in vivo* expansion of regulatory T cells could protect against cerebral ischemic injury (Li et al., 2013b; Zhang et al., 2018) and meanwhile preserve the immune homeostasis after stroke (Li et al., 2013b). However, the immune suppressive regulatory T cells may be attracted to the tumor via the VEGF-neuropilin 1 signaling when the stroke recipient is complicated by cancer (Wang L. et al., 2018). Other critical issues related to immune cell-based therapy include availability or preparation of immune cells, major histocompatibility complex molecules which may initiate immune responses, preservation of purified immune cells and et al., all of which hinder the clinical translation of immune cell-based therapies.

### Mitochondrial Dynamic of Myeloid Cells as Therapeutic Target

Mitochondria, a highly dynamic cellular organelle that plays pivotal role in metabolism and apoptosis is also deeply involved in the interplay between cellular metabolism and both innate and adaptive immune responses (Tur et al., 2017). They govern the dependence of cellular metabolism and the coordination of the tricarboxylic acid (TCA) cycle to modulate oxidative phosphorylation rates and adenosine triphosphate (ATP) production, as well as the important intermediates to support other metabolic processes (Tur et al., 2017). Therapeutically restoring mitochondrial function can reprogram inflammatory macrophages into anti-inflammatory cells by regulating cellular metabolic pathways (Van den Bossche et al., 2016). Alternatively activated or naive macrophages predominantly use oxidative phosphorylation for their energy support, whereas the aerobic glycolysis and pentose phosphate pathway can be robustly enhanced in classically activated macrophages (Liu and Ho, 2018), with significantly increased glucose uptake. Whereas the fatty acids uptake is enhanced to support anti-inflammatory microglia/macrophage polarization and functions (Van den Bossche et al., 2016). As the central regulator of cellular metabolism, mitochondria are emerging as a pivotal therapeutic target to regulate the phenotype polarization of microglia/macrophages (Mehta et al., 2017). After cerebral ischemic stroke, enhanced glycolysis and fatty acid synthesis have been found in peripheral T cells, with increased expression of acetyl-coenzyme A (Wang et al., 2019). Hyper-glycolysis can induce the key glycolytic enzyme hexokinase 2 (HK2), which further results in accumulated acetyl-coenzyme A and subsequent histone acetylation and transcriptional activation of interleukin (IL)-1β and over-activation of the microglia-mediated neuroinflammation after cerebral ischemic stroke (Li et al., 2018a). However, it still should be kept in mind that the phenotypes of microglia/macrophages are far more complicated than pro-inflammatory or anti-inflammatory as we mentioned above. How does the mitochondria function and the metabolic process affect other phenotypes still remains largely unknown.

### Immunological Effects of Extracellular Vesicles (EVs) on Ischemic Stroke

Extracellular vesicles (EVs) which can be released by almost all cell types can appear in all body fluids as membrane-surrounded vesicles. There are two distinct types of EV according to their origin: shedding microvesicles apoptotic bodies and exosomes. Because exosomes are able to cross the BBB and the blood-cerebrospinal fluid barrier, it allows crosstalk between the periphery and the brain. On one hand, damaged endothelium releases pro-inflammatory factors and EVs that pass through the compromised BBB after stroke. These EVs can activate microglia and astrocytes to release pro-inflammatory cytokines (TNFα, IL1β) (Zagrean et al., 2018). On the other hand, exosomes are emerging as a powerful diagnostic tool and a promising therapeutic shuttle of natural nanoparticles (Zagrean et al., 2018). It has been suggested that microglia-derived exosomes may contain microRNAs (miR-124-3p) that can inhibit neuronal inflammation and promote neurite outgrowth by transferring into neurons after traumatic brain injury (Ponomarev et al., 2013; Huang et al., 2018). In addition, intravenous administration of macrophage exosomes can cross the BBB which serve as natural nanocarriers to deliver a cargo protein, the brain derived neurotrophic factor to the injured brain (Yuan et al., 2017). Treatment with exosomes secreted from LPS-stimulated macrophage or MNCs has been shown to reduce brain infarct volume and improve neurological function after focal cerebral ischemic stroke (Altmann et al., 2014; Zheng et al., 2019). In addition, exosomes derived from several different cell types, such as mesenchymal stromal cells, adipose-derived stem cells (ADSCs), embryonic stem cells (ESCs) and neural stem cells (NSCs) (Rufino-Ramos et al., 2017; Cunningham et al., 2018; Webb et al., 2018), and exosomes from these cells have been shown to reduce ischemic volume, promote white matter repair and help neurovascular restoration following ischemia-reperfusion injury (Xin et al., 2013; Otero-Ortega et al., 2017; Jiang et al., 2018; Venkat et al., 2018; Webb et al., 2018). Therefore, it’s reasonable to envision that the exosome from the “healing” type of myeloid cells holds the promise to serve as an intriguing therapeutic strategy for future stroke therapy.

## Concluding Remarks and Future Perspectives

As highlighted in this review, myeloid cells play an important role in tissue remodeling during the late stage of stroke via pleiotropic mechanisms, such as inhibiting immune inflammation, devouring harmful factors, removing tissue fragments, secreting neurotrophic substances, et al. Scavenger receptors such as Msf1, CD36, TREM2, CRs, MRs and MerTK all contribute to the clearing of necrotic debris and set the stage for NVU remodeling in the repair phase. Several mechanisms are involved in their anti- inflammatory functions to turn into potential protective phenotype through secreting anti-inflammatory cytokines such as IL-10 and TGF-β. With their “healing” effects during neurovascular renewal, myeloid cells are promising to provide novel therapeutic targets (Table 1). Meanwhile, myeloid cell-based therapy, such as bone marrow derived myeloid stem cell perfusion, mitochondrial therapy and EVs-based therapy are also gaining increasing attention for the stroke treatment. However, there are myriad unknown areas that warrant further investigation to foster novel therapeutic strategies, such as the complex mix of “help me” signals that initiate the activation and functional switching of myeloid cells, the interactions between the NVU repair processes and myeloid cells and the underlying mechanisms. As our understanding of the role of myeloid cells in the post-stroke NVU repair grows, we may have the potential to design better myeloid cell targeted treatments to improve the neurological outcome of stroke patients.

**TABLE 1:** Potential immunotherapeutic targets on myeloid cells for NVU repair after stroke.

**Cellular targets**	**Functions and underlying mechanisms**	**References**
Msr1	Clearance of DAMPs; reduce the expression of inflammatory cytokines	Shichita et al., 2017
CD36	Clearance of cell debris; attenuation of nuclear factor-κB mediated inflammation;	Cho et al., 2005; Woo et al., 2012
TREM2	Promotes microglial survival by inhibiting β-catenin degradation *via* the Akt/GSK3β signaling pathway; phagocytosis apoptotic neurons, amyloid plaques and anionic and zwitterionic lipids on damaged neurons	Hsieh et al., 2009; Wang et al., 2015; Yuan et al., 2016; Zheng et al., 2017
CR1	Induction of phagocytosis by interacting with its ligands; clearing debris, apoptotic or necrotic neurons and limiting neuroinflammation	Rotshenker, 2003; Fourgeaud and Boulanger, 2007
MRs	Enhance the clearance ability of microglia/macrophages	Giraldi-Guimaraes et al., 2012
MerTK	Phagocytosis of ischemic neurons; clearance of hetatoma and reduce iron deposition	Neher et al., 2013; Chang et al., 2018
arginase-1, CD206, Ym1 and TGF β and IL-10	Induce the anti-inflammatory effect of microglia and macrophages by activating CX3CL1-CX3CR1 signaling, IL-33/ST2 signaling and neuronal IL-4, PPARγ	Mizuno et al., 2003; Lyons et al., 2009; Zhao et al., 2015; Liu X. et al., 2016; Yang et al., 2017
PPARγ in N2 neutrophils	Limit excessive immune response, promoting neuronal survival and successful brain remodeling after ischemic stroke and ICH	Hermann et al., 2018
lactoferrin	Iron-scavenging and clearance of hetatoma	Zhao X. et al., 2017
IGF-1	Promote proliferation and differentiation of NSCs	Thored et al., 2009
BDNF	Regulate synaptic function and promote multiple learning tasks performance	Coull et al., 2005
IL-8, VEGF, TGF-β, prostaglandin, MMP-9	Enhance the angiogenic process	Sanberg et al., 2010
Filopodia or lamellipodia	Physically adhere to both endothelial ends at the cerebral vascular rupture lesion	Liu C. et al., 2016
iron-releasing phenotype of microglia	Promoting oligodendrocyte progenitor cell differentiation	Shin et al., 2018
EVs, exosomes, miRNA124-3p	Inhibit neuronal inflammation and promote neurite outgrowth	Ponomarev et al., 2013; Huang et al., 2018

*NVU, neurovascular unit; Msr1, macrophage scavenger receptor 1; DAMP, damage associated molecular patterns; CD36, cluster of differentiation 36; TREM2, triggering receptor expressed on myeloid cells-2; CR, complement receptor; MR, mannose receptor; MerTK, mer receptor tyrosine kinase; PPARγ, peroxisome proliferator-activated receptor γ; CX3CL1-CX3CR1, chemokine (C-X3-C motif) ligand 1-CX3C chemokine receptor 1; IL-33, interleukin 33; IL-4, interleukin 4; ICH, intracerebral hemorrhage; IGF-1, insulin-like growth factor-1; NSC, neural stem cell; BDNF, brain-derived neurotrophic factor; IL-8, interleukin 8; VEGF, vascular endothelial growth factor; TGF-β, transforming growth factor β; MMP-9, matrix metallopeptidase 9; EVs, extracellular vesicles.*

## Author Contributions

All authors listed have made a substantial, direct and intellectual contribution to the work, and approved it for publication.

## Conflict of Interest Statement

The authors declare that the research was conducted in the absence of any commercial or financial relationships that could be construed as a potential conflict of interest.
